# Female chronotype is not related to annual and lifetime reproductive success in a free-living songbird

**DOI:** 10.1098/rsos.250380

**Published:** 2025-09-03

**Authors:** Marjolein Meijdam, Marcel Eens, Wendt Müller

**Affiliations:** ^1^ Department of Biology, University of Antwerp, Antwerp, Belgium; ^2^ Department of Biology, Ethology Group, University of Antwerp, Antwerp, Belgium; ^3^ Department of Biology, University of Antwerp, Antwerp, Belgium

**Keywords:** chronotype, fitness, longevity, reproductive success

## Abstract

Circadian clocks play a crucial role in regulating the sleep–wake rhythm of organisms, aligning their activity with fluctuating environmental factors, such as light intensity. Still, significant and consistent interindividual differences in the timing of activity, known as chronotypes, have been observed across various species, but whether this affects fitness is still unknown. While previous studies have primarily focused on annual reproductive success, few studies have examined associations between chronotype and lifetime reproductive success. Here, we, therefore, study the association between chronotype, i.e. the emergence time from the nest box in the morning at the onset of reproduction, and annual reproductive success, lifetime reproductive success and longevity in free-living female great tits. We used a long-term dataset of individually marked birds, with the number of eggs, fledglings and recruits produced by a female serving as proxies for reproductive success and the age at death as a measure of longevity. Intriguingly, we did not find significant relationships between chronotype and reproductive success or longevity, and hence, no evidence for directional selection on chronotypes. As we found neither evidence of stabilizing nor disruptive selection, we could not show evolutionary implications of individual variation in chronotypes. Further experimental investigations and complementary studies in other populations will be necessary to understand whether and to what extent chronotypes are adaptive and whether our results are generalizable.

## 1. Introduction

Prominent abiotic and biotic factors in the environment, such as the light conditions, temperature, food availability and the presence or absence of predators follow a daily cycle [1]. Animals can synchronize their behaviour, such as the timing of activity, with these cycles via biological clocks, which provide a self-sustaining circadian oscillation of about 24 h at the molecular level that is sensitive to the environment. Biological clocks are ubiquitous and are considered to be functionally adaptive, as they may enable organisms to optimize their use of the environment [2,3]. Intriguingly, within species, there are often remarkable differences between individuals in the behavioural output of the circadian clock, i.e. the timing of activity onset and offset [4–12]. An individual’s temporal phenotype is typically called chronotype. Individual differences in chronotype may be caused by variation in the endogenous circadian period (τ), which is the duration of one full cycle of the circadian rhythm in the absence of any time giving cues. τ often slightly deviates from 24 h [13–16]. Individuals with relatively short cycle lengths have earlier chronotypes, such individuals generally time their activity earlier in the day, and vice versa. In free-living species where establishing the chronotype is difficult to achieve, the timing of activity is typically taken as a measure of the chronotype [4,6,8]. This behavioural-ecological definition differs from the human literature where it refers to the midpoint of sleep (e.g. [17]).

Having an early or late chronotype can have important functional consequences in the wild. Starting activity earlier during the day relative to conspecifics may increase feeding opportunities and daily food intake [18], increase survival [1,19] and could also affect parental provisioning during the nestling period [20]. Furthermore, an early onset of activity might also be beneficial in the context of reproduction. In many bird species, courtship occurs during a specific daily time window, typically early in the day [21]. However, early rising may also come with certain costs. For example, early rising individuals may face greater predation risk due to limited vision, or they could incur energetic costs due to low temperatures in the morning [1]. These opportunities and risks must be weighed against each other. Potentially, a specific starting time of activity exists that optimizes the balance of opportunities and risks, maximizing fitness. As the cost–benefit balance of a specific timing can also depend on the timing of other individuals, frequency-dependent selection may also play a crucial role in maintaining variation in chronotypes [22]. Furthermore, selection pressures may differ depending on the environment in which an individual lives, leading to spatially or temporally fluctuating selection pressures [23].

So far, studies in the wild have investigated a number of fitness-relevant aspects, such as the reproductive consequences of variation in chronotype, showing that the timing of the dawn song in male birds is related to extra-pair paternity ([24–28], but see [29]). Furthermore, female European starlings (*Sturnus vulgaris*) that initiated a second brood had earlier activity onset during the chick-rearing period [8]. However, no other measures of annual reproductive success, like clutch size and number of fledglings, were affected by chronotype [8]. In females, blue tits (*Cyanistes caeruleus*) and great tits (*Parus major*) chronotype was not related to annual reproductive success [28,30], although a more recent study found that female great tits with earlier chronotypes had more fledglings [31]. Current empirical evidence on potential fitness consequences of chronotype, particularly in females, is thus mixed. Importantly, it has been neglected that fitness is not only affected by reproductive success within a given year, at least in more long-lived species, but also by survival and hence future breeding opportunities, which have not been taken into account thus far. Including survival and studying lifetime reproductive success instead of annual reproductive success may hence be crucial to increase our understanding of the effect that chronotype may have on fitness [32]. It may reveal new insights into the adaptive functions of individual variation in chronotypes.

In this study, we investigated the consequences of differences in the average timing of activity onset in the morning (i.e. chronotype) for reproduction and survival in female great tits breeding in a suburban population. We used a large long-term dataset, for which we have shown both short- and long-term repeatable variation in the timing of activity onset at the onset of egg laying [9]. This has recently been shown to be indicative of the timing of activities across a large part of the reproductive period [20]. Our long-term dataset enabled investigating correlations between the timing of activity onset and fitness both at the among- and within-individual level. The timing of activity onset in the morning was determined by measuring the emergence time from the nest box. Furthermore, we did examine not only annual reproductive success (i.e. annual number of eggs and annual number of fledglings) but also lifetime reproductive success (i.e. lifetime number of eggs, lifetime number of fledglings, lifetime number of recruits). We used longevity (i.e. age at death) as a measure of survival. Finally, in addition to directional selection, we also investigated stabilizing or disruptive selection, i.e. whether individuals with emergence times closer to or more deviant from the population mean are more successful.

## 2. Methods

This study was carried out in a suburban nest box breeding great tit population in Antwerp, Belgium (51°09′46.1″ N, 4°24′13.3″ E), that has been monitored since 1995 [33]. Each year (1995–2023), individuals were captured when roosting in the nest box in winter, during which their age (yearling or older) and sex were determined based on plumage characteristics. To facilitate individual recognition, individuals received a metal ring, two coloured rings and a passive integrated transponder (PIT) tag around their tarsi. Individuals not captured during winter were instead caught on the nest during the chick rearing phase of the breeding season, when the chicks were at least 8 days old. Chicks were ringed when between 9 and 14 days old. We routinely determined clutch initiation dates, clutch sizes and the number of fledglings since 1997 [33–35]. In our population, second clutches occur and clutch sizes and the number of fledglings were determined as for the first clutch.

### 2.1. Emergence time

In 2018, 2019, 2020 and 2021, female activity onset in the morning was repeatedly recorded during the egg-laying period, by measuring the emergence time from the nest box in the morning. We mainly used SongMeters (*n* = 1427; SongMeterTM SM2+; Wildlife Acoustics, Inc., USA), complemented by radio frequency identification (RFID) loggers (*n* = 57; EM4102 data logger; Eccel Technology Ltd, Aylesbury, UK) and cameras (*n* = 5; Pakatak PAK-MIR5; Essex, UK) if insufficient SongMeters were available. SongMeters were positioned on top of the nest box, with one microphone inside and the other microphone outside the nest box. Recording sessions were conducted from 04.00 to 08.00 central European time (CET) during the wintertime period, and after the transition to summertime recordings took place from 03.00 to 08.00 CET. Upon the females’ departure from the nest box in the morning, the sound of their wings, their claws on the nest box [36] and the female passing through the opening of the nest box can be heard. Emergence time was determined using Avisoft SASLab Pro 5.2.14 [37]. RFID readers registered PIT-tagged individuals upon passing through the two antennas positioned around the nest box opening. The PIT tag number and the corresponding entry and exit times were recorded (for more details, see [38]). Infrared-sensitive cameras, mounted under the nest box lid and facing downward, started recording immediately after installation at least 2 h before sunset and were collected the following morning at least 2 h after sunrise [35]. Throughout the 2018 breeding season, emergence times were often simultaneously measured with both RFID loggers and SongMeters. On average, there was a 1.5 ± 0.4 min difference between the two methods (mean ± standard error; *n* = 633 mornings recorded at 74 nest boxes [39]). We determined emergence times relative to sunrise (negative = before sunrise, positive = after sunrise), and hereafter, emergence time always concerns relative times. Temperature data were retrieved via: https://www.wunderground.com/history/daily/be/antwerp. In total, we measured 1489 emergence times in 297 females, of which 47 individuals were removed from the dataset. The identity of these birds was unknown due to nest failure before identification (*n* = 26) or because they were already older than 1 year upon first capture (*n* = 21) and so their age could not be accurately estimated (see table 1 for sample sizes per year; sample size for analysis *n* = 250 females). Emergence times were determined in all 4 years for seven individuals, in 3 years for 22 individuals, in 2 years for 64 individuals and in only 1 year for 157 individuals. In a previous study, we have shown in this study population that emergence time is repeatable both in the short and long term [9].

**Table 1.** Emergence time sample sizes per year after removal of 47 individuals with unknown identity and/or age.

year	*n*_females_	mean number of measurements per female	s.d. of the number of measurements per female
2018	106	3.12	1.08
2019	113	2.96	1.09
2020	103	3.89	1.10
2021	57	4.42	1.05

### 2.2. Reproduction and survival

For all individuals whose emergence time was recorded, we assessed annual reproductive success, lifetime reproductive success and longevity. Annual reproductive success was determined by the annual number of eggs (i.e. the sum of the number of eggs in the first and second clutches in a given year) and the annual number of fledglings (i.e. the sum of the number of fledglings in the first and second clutches in a given year). The annual number of eggs was not known in 1 year for seven individuals and the number of fledglings was not known in 1 year for 10 individuals. So for a total of 11 females, 1 year of reproductive data was missing, so these were excluded from the lifetime reproductive success analyses (final samples size *n* = 226 females). Lifetime reproductive success was determined by the lifetime number of eggs, lifetime number of fledglings and lifetime number of recruits (i.e. offspring recruited into the breeding population). As emergence times were not necessarily recorded in the first year of breeding, data on reproductive success collected in years prior to 2018 were also incorporated into our measure of lifetime reproductive success. Longevity was determined by the age at death, defined as the age at which individuals were last breeding in the population. Individuals considered dead were also not observed outside of the breeding season. Some of these individuals may have dispersed out of the breeding population, but as the number of adult immigrants was relatively low (*n* = 21 on 297 females) and the number of breeding pairs in the population is stable, we expect this was only a minor proportion. We excluded 13 individuals from the dataset that were still alive at the moment of data analyses in autumn 2023, i.e. last seen in the 2023 breeding season, which represents the end of data collection (also for recruitment).

### 2.3. Statistical analyses

All statistical analyses were performed in R 4.1.3 [40]. The MCMCglmm package (v. 2.35) was used throughout to fit multivariate mixed models [41]. Emergence time was scaled to unit variance prior to all analyses. Reproductive success and longevity variables were divided by the population mean to obtain relative values.

To study the covariation between the annual reproductive success and emergence time, we first constructed two bivariate mixed models. Such models use the explanatory variables for each response variable separately and investigate the relationship between the two response variables while taking the effects of the explanatory variables into account. The first bivariate model included both the relative annual number of eggs and emergence time from the nest box as response variables ([42]; *n* = 243). Year (2018, 2019, 2020 or 2021) and age (1–6 years) were included as fixed effects for both variables (table 2). For emergence time, we included a polynomial date effect (mean-centred within years) using the poly() function up to the second order and the temperature at sunrise (mean-centred within years [10,43,44]). At the level of the relative annual number of eggs, we included the clutch initiation date (mean-centred within years). Female identity was included as a random effect, which enabled partitioning the (co)variance into the among- and within-individual components [45]. As the values of the covariances depend strongly on the variables that were included in the model, they do not give much information on the strength of the relationship, only on the direction. For easier interpretation, the among- and within-individual covariance between relative annual number of eggs and emergence time were therefore converted into correlations by dividing the respective covariance by the square root of the product of the respective variances [42]. A second bivariate mixed model was constructed to study the covariance between emergence time and the relative annual number of fledglings (*n* = 240). This model included the same fixed and random effects as described above (table 2). We repeated these analyses using only the number of eggs and fledglings from first clutches, as second clutches may also represent replacement clutches, as for clutches that are lost at an early stage the identity of the female is not always known (for the results, see electronic supplementary material, tables S1 and S2).

**Table 2.** Overview of fixed effects included in the different bivariate models.

response variables	fixed effects					
	year	age	date	date 2	temperature	lay date
emergence time (1) and number of eggs (annual) (2)	1 and 2	1 and 2	1	1	1	2
emergence time (1) and number of fledglings (annual) (2)	1 and 2	1 and 2	1	1	1	2
emergence time (1) and number of eggs (lifetime) (2)	1	1	1	1	1	—
emergence time (1) and number of fledglings (lifetime) (2)	1	1	1	1	1	—
emergence time (1) and number of recruits (lifetime) (2)	1	1	1	1	1	—
emergence time (1) and age at death (2)	1	1	1	1	1	—
emergence time (deviation from population mean) (1) and number of eggs (annual) (2)	1 and 2	1 and 2	1	1	1	2
emergence time (deviation from population mean) (1) and number of fledglings (annual) (2)	1 and 2	1 and 2	1	1	1	2
emergence time (deviation from population mean) (1) and number of eggs (lifetime) (2)	1	1	1	1	1	—
emergence time (deviation from population mean) (1) and number of fledglings (lifetime) (2)	1	1	1	1	1	—
emergence time (deviation from population mean) (1) and number of recruits (lifetime) (2)	1	1	1	1	1	—
emergence time (deviation from population mean) (1) and age at death (2)	1	1	1	1	1	—

We constructed four bivariate models to study the covariation between chronotype and lifetime reproductive success that included emergence time and either relative lifetime number of eggs, relative lifetime number of fledglings, relative lifetime number of recruits or relative age at death as response variables. These models included year (2018, 2019, 2020 or 2021), age (in years), a polynomial date effect (mean-centred within years) up to the second order and the temperature at sunrise (mean-centred within years) as fixed effects at the level of emergence time (table 2). Again, female identity was included as random effect. Within-individual variances were fixed at 0.0001 for all lifetime reproductive success parameters and age at death (see electronic supplementary material for the details on prior specifications).

To examine whether stabilizing or disruptive selection was acting on emergence time we determined whether individuals with emergence times closer to the population mean had higher reproductive success and longer longevity than individuals with more extreme emergence times. Therefore, for each observation, we calculated the absolute deviation from the population mean for emergence time. Then, we ran bivariate mixed models with the absolute deviation from the population mean for emergence time (square root transformed) and either annual number of eggs, annual number of fledglings, lifetime number of eggs, lifetime number of fledglings, lifetime number of recruits or age at death as response variables. We included the same fixed and random effects as described above (table 2).

All multivariate models were run with Gaussian error distributions. We set the number of iterations at 420 000, the burn-in phase at 20 000 and the thinning interval at 200. For the models including the annual number of fledglings, we increased the iterations to 550 000, the burn-in phase to 50 000 and the thinning interval to 250 due to convergence issues. For the model that included the absolute deviation from the population mean for emergence time and age at death as explanatory variables, we increased the number of iterations to 850 000, the burn-in phase to 50 000 and the thinning interval to 400. The results presented are from models with a non-informative parameter expanded prior (see electronic supplementary material for prior specifications). The use of alternative prior specifications (i.e. inverse Wishart and inverse gamma) gave qualitatively similar results. Traces of posterior distributions were checked visually, and autocorrelation between successively stored iterations was less than 0.1 in all cases [41]. Model convergence and mixing were assessed using Gelman–Rubin statistics between chains (i.e. the potential scale reduction factor was less than 1.1 in all cases [46]). Results presented are posterior mean estimates with associated 95% credible intervals (CrI), unless stated otherwise. Fixed-effects and correlation estimates were considered to find strong support if the 95% CrI did not overlap with zero.

## 3. Results

Emergence time ranged from 127 min before sunrise up to 91 min after sunrise (mean ± s.d. = 8.45 ± 17.67, median = 7.31) and was significantly affected by date, yet in a nonlinear way (table 3). Across the breeding season, a later initiation of egg laying was associated with progressively later emergence from the nest box and this effect became stronger towards the end of the breeding season. As the season progressed, emergence times also started to deviate more from the population mean (table 4). On colder mornings, females emerged later from the nest box, but temperature at sunrise did not affect the deviation of the emergence time from the population mean (table 4). There was variation among years in emergence times, with earlier emergence times in 2020 compared with 2018. In 2019, emergence times were closest to the population mean. Furthermore, emergence times advanced with age (table 3), but age did not affect the deviation of the emergence time from the population mean (table 4).

**Table 3.** Results from a bivariate mixed model with emergence time from the nest box (in minutes relative to sunrise) and the relative annual number of eggs as response variables. Estimates of fixed (β) and random (σ²) components are shown with 95% CrI and fixed effects that found strong support are presented in bold.

	emergence time	relative annual number of eggs
*fixed effects*		
intercept	**0.353 (0.179 to 0.495**)	**0.880 (0.797 to 0.952**)
date^a^	**9.968 (6.604 to 13.451**)	—
date 2^a^	**3.645 (0.858 to 6.769**)	—
temperature at sunrise^b^	**−0.031 (−0.046 to −0.016**)	—
year 2019^c^	−0.092 (−0.240 to 0.061)	**0.112 (0.030 to 0.189**)
year 2020^c^	**−0.305 (−0.498 to −0.132**)	0.033 (−0.052 to 0.110)
year 2021^c^	0.211 (−0.025 to 0.470)	0.060 (−0.038 to 0.173)
age	**−0.126 (−0.203 to −0.037**)	**0.034 (0.000 to 0.069**)
clutch initiation date^d^	—	**−0.017 (−0.022 to −0.011**)
*random effects*		
femaleID	0.486 (0.386 to 0.596)	0.019 (0.004 to 0.036)
residual	0.487 (0.444 to 0.529)	0.079 (0.062 to 0.097)

^a^ Date of measurement mean-centred within years.

^b^ Temperature at sunrise mean-centred within years.

^c^ 2018 is used as reference year.

^d^ Clutch initiation date mean-centred within years.

**Table 4.** Results from a bivariate mixed model with the absolute deviation from the population mean for emergence time (in minutes relative to sunrise) and the relative annual number of eggs as response variables. Estimates of fixed (β) and random (σ²) components are shown with 95% CrI and fixed effects that found strong support are presented in bold.

	emergence time (deviation from population mean)	relative annual number of eggs
*fixed effects*		
intercept	−0.04 (−0.20 to 0.11)	**0.88 (0.80 to 0.96**)
date^a^	**4.23 (0.72 to 7.66**)	—
date 2^a^	1.59 (−1.54 to 5.03)	—
temperature at sunrise^b^	−0.01 (−0.02 to 0.01)	—
year 2019^c^	**−0.17 (−0.33 to −0.01**)	**0.11 (0.03 to 0.19**)
year 2020^c^	0.10 (−0.07 to 0.27)	0.03 (−0.05 to 0.11)
year 2021^c^	0.17 (−0.05 to 0.39)	0.06 (−0.04 to 0.17)
age	0.01 (−0.06 to 0.09)	**0.03 (0.00 to 0.07**)
clutch initiation date^d^	—	**−0.017 (−0.023 to −0.012**)
*random effects*		
femaleID	0.34 (0.25 to 0.43)	0.02 (0.00 to 0.03)
residual	0.68 (0.63 to 0.75)	0.08 (0.06 to 0.09)

^a^ Date of measurement mean-centred within years.

^b^ Temperature at sunrise mean-centred within years.

^c^ 2018 is used as reference year.

^d^ Clutch initiation date mean-centred within years.

The annual number of eggs ranged from 3 up to 19 (mean ± s.d. = 9.38 ± 3.06, median = 9, *n* = 243) and the annual number of fledglings ranged from 0 up to 15 (mean ± s.d. = 6.61 ± 3.38, median = 7, *n* = 240). In 2019, the annual number of eggs was significantly higher than in 2018 (tables 3 and 4) and the number of fledglings was lower in 2020 and 2021 compared with 2018 (estimate_2019_ = 0.03 [−0.10 to 0.15], estimate_2020_ = −0.30 [−0.44 to −0.17], estimate_2021_ = −0.20 [−0.36 to −0.04]). The annual number of eggs and fledglings was lower for later clutch initiation dates (tables 3 and 4; Estimate_annual number of fledglings_ = −0.02 [−0.03 to −0.01]). Finally, the annual number of eggs increased with age, while the annual number of fledglings did not (tables 3 and 4; Estimate_annual number of fledglings_ = −0.002 [−0.057 to 0.048]). When only first clutches were considered for analysis, the year and age effects on the annual number of eggs and the effect of clutch initiation date on the annual number of fledglings disappeared (electronic supplementary material, table S1).

The lifetime number of eggs ranged from 4 up to 68 (mean ± s.d. = 19.29 ± 14.05, median = 13.5, *n* = 226), the lifetime number of fledglings from 0 up to 60 (mean ± s.d. = 13.03 ± 9.98, median = 9, *n* = 226), the lifetime number of recruits from 0 up to 10 (mean ± s.d. = 0.88 ± 1.38, median = 0, *n* = 226) and age at death from 1 up to 7 (mean ± s.d. = 2.07 ± 1.31, median = 2, *n* = 226).

**Figure 1.**
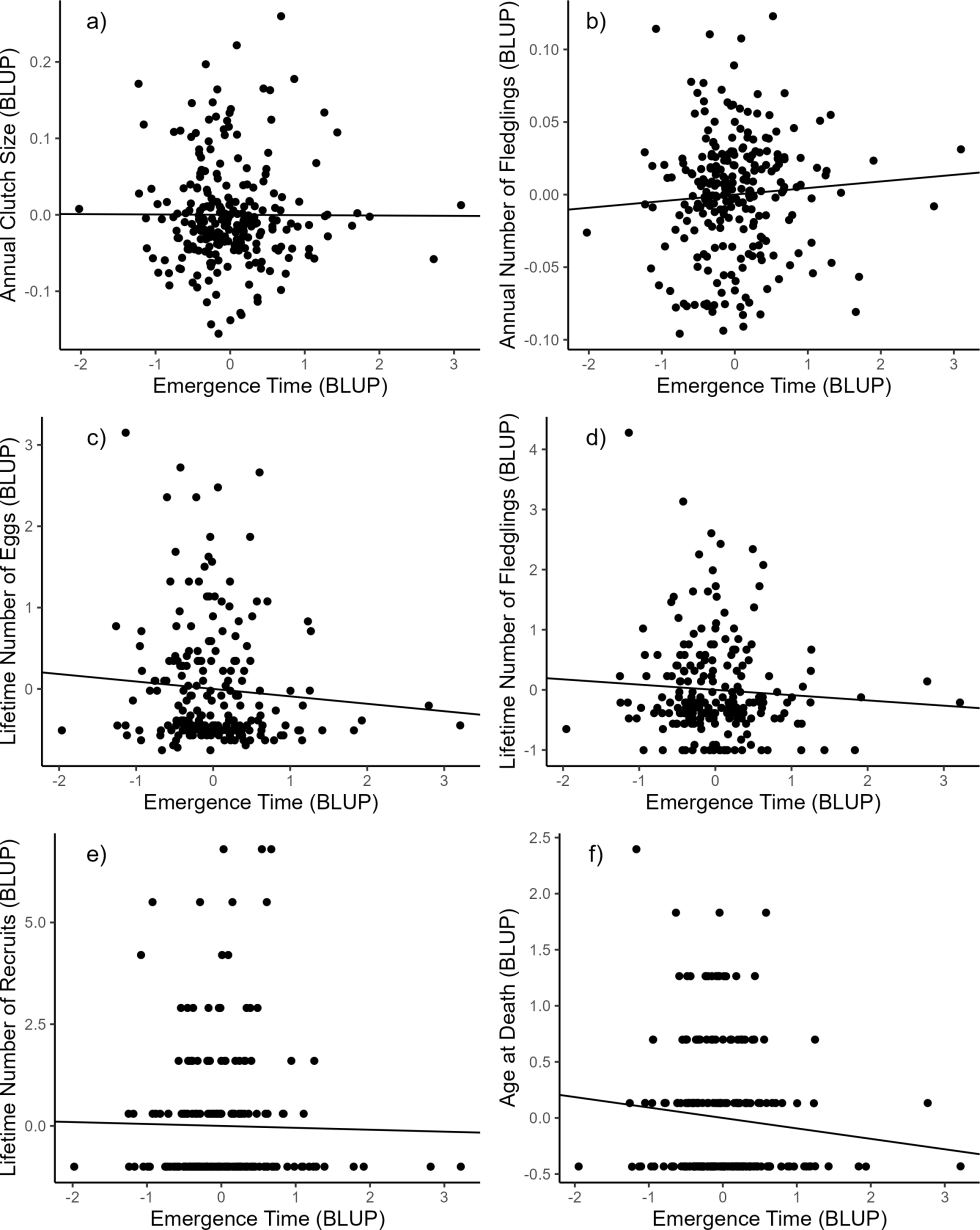
Among-individual correlations between emergence time from the nest box and (a) the annual clutch size, (b) the annual number of fledglings, (c) the lifetime number of eggs, (d) the lifetime number of fledglings, (e) the lifetime number of recruits and (f) the age at death in female great tits. Best linear unbiased predictions (BLUPs) were extracted from bivariate mixed models (table 5) and used here for illustrative purposes only [42]. All reproductive variables and the age at death were divided by the population mean. All correlations were not significant.

**Figure 2.**
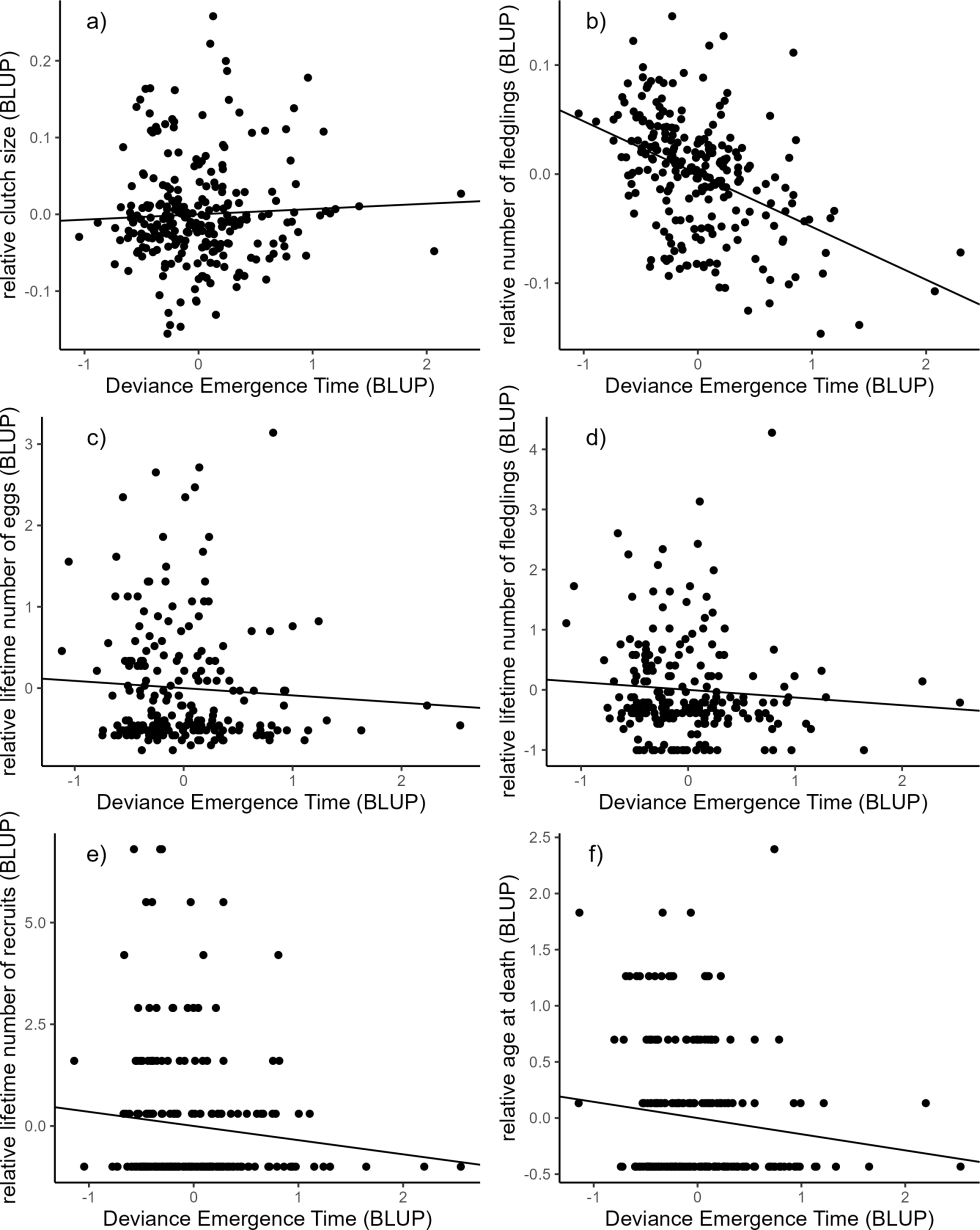
Among-individual correlations between the absolute deviance of the emergence time from the population mean and (a) the annual clutch size, (b) the annual number of fledglings, (c) the lifetime number of eggs, (d) the lifetime number of fledglings, (e) the lifetime number of recruits and (f) the age at death in female great tits. Best linear unbiased predictions (BLUPs) were extracted from bivariate mixed models (table 5) and used here for illustrative purposes only [42]. All reproductive variables and the age at death were divided by the population mean. All correlations were not significant.

Emergence time was not significantly correlated with any of the annual or lifetime reproductive success parameters nor with longevity, either at the among- or within-individual level (table 5; figure 1). The absolute deviance of the emergence time from the population mean was also not associated with reproductive success or longevity (table 5; figure 2). Individuals with emergence times closer to the population mean were thus not more successful than individuals with more extreme emergence times. Only including first clutches for the annual number of eggs and the annual number of fledglings did not change these results (electronic supplementary material, table S2).

**Table 5.** Covariances and correlations (with 95% credible intervals) regarding emergence time from the nest box in the morning and the absolute deviation from the population mean for emergence time in relation to reproductive success and age at death at the among- and within-individual level.

*response variables*	covariance among individuals	correlation among individuals	covariance within individuals	correlation within individuals
emergence time and number of eggs (annual)	0.00 (−0.02 to 0.02)	−0.01 (−0.32 to 0.28)	0.00 (−0.03 to 0.03)	0.00 (−0.11 to 0.11)
emergence time and number of fledglings (annual)	0.00 (−0.03 to 0.04)	0.03 (−0.54 to 0.78)	0.01 (−0.02 to 0.05)	0.04 (−0.06 to 0.15)
emergence time and number of eggs (lifetime)	−0.06 (−0.15 to 0.03)	−0.12 (−0.28 to 0.05)	—	—
emergence time and number of fledglings (lifetime)	−0.04 (−0.14 to 0.05)	−0.07 (−0.24 to 0.09)	—	—
emergence time and number of recruits (lifetime)	−0.02 (−0.20 to 0.17)	−0.01 (−0.17 to 0.15)	—	—
emergence time and age at death	−0.05 (−0.13 to 0.02)	−0.13 (−0.30 to 0.03)	—	—
emergence time (deviation from population mean) and number of eggs (annual)	0.00 (−0.024 to 0.025)	0.02 (−0.29 to 0.40)	0.01 (−0.02 to 0.03)	0.07 (−0.05 to 0.20)
emergence time (deviation from population mean) and number of fledglings (annual)	−0.02 (−0.05 to 0.02)	−0.23 (−0.86 to 0.34)	−0.01 (−0.05 to 0.03)	−0.01 (−0.13 to 0.09)
emergence time (deviation from population mean) and number of eggs (lifetime)	−0.03 (−0.12 to 0.05)	−0.06 (−0.23 to 0.12)	—	—
emergence time (deviation from population mean) and number of fledglings (lifetime)	−0.05 (−0.14 to 0.034)	−0.10 (−0.28 to 0.07)	—	—
emergence time (deviation from population mean) and number of recruits (lifetime)	−0.12 (−0.29 to 0.06)	−0.12 (−0.28 to 0.05)	—	—
emergence time (deviation from population mean) and age at death	−0.06 (−0.12 to 0.02)	−0.15 (−0.32 to 0.04)	—	—

## 4. Discussion

Individual variation in the timing of activity onset, which in free-living species is typically used as a measure of the chronotype, is increasingly studied in the wild, but we still lack a proper understanding of its adaptive significance. Using a long-term dataset allowed us to investigate associations between chronotype (i.e. emergence time) and longevity and ultimately lifetime reproductive success. This has, to the best of our knowledge, never been done in free-living animals, but it could be more closely linked to an individual’s fitness than fitness proxies such as annual reproductive success as have been used in most previous studies. However, we found no significant relationships between chronotype and (different measures of) fitness. Below we discuss the possible causes of why chronotypes are not linked to fitness outputs as well as possible eco-evolutionary implications of our findings.

### 4.1. Timing of activity onset, reproductive success and longevity

Our study, which was based on a substantial, long-term dataset of individually marked great tits, did not provide any evidence that emergence time, a repeatable measure of chronotype, is related to annual reproductive success. This finding is, among others, different from the outcome of a recent study by Womack *et al*. [31], who found that female great tits with an earlier onset of activity in the morning raised more fledglings. This was found in both urban and rural populations. So even though these habitats are known for phenotypic divergence (e.g. [47,48]), it is unlikely that the differences between studies are due to habitat, i.e. our population is situated in a suburban environment. However, selection pressures on chronotype may in general differ between populations as they might vary with environmental factors, which were not explicitly considered in either of the studies. Nevertheless, our results were in agreement with studies in European starlings (no effect of onset of activity on cumulative 2 year productivity [8]) and in blue tits (no effect of onset of activity on lay date or clutch size [28]). Thus, at present, across species evidence rather indicates that individual variation in emergence time does not relate to reproductive success. This is further supported by the fact that we did not find any effects of the timing of activity onset on lifetime reproductive success, nor was there any effect on longevity, as could have been expected in a trade-off framework. Chronotype was thus not subjected to directional selection, at least in females. Despite the fact that our outcome is very closely linked to an individual’s fitness as we could analyse a long-term, extensive database, there are limitations. It has to be taken into account that in the case of the lifetime number of recruits, we used a minimum estimate, as we only counted local recruits and could not determine emigration rates [32,49]. Given the spatial limitations of our study population, the results for the lifetime number of recruits should thus be interpreted with caution.

### 4.2. Lack of selection on emergence time

While there was no evidence for directional selection on chronotypes, we also did not find indications for stabilizing or disruptive selection. Individuals with emergence times closer to the population mean were equally successful as individuals with more extreme emergence times, i.e. very early (up to 120 min before sunrise) or very late chronotypes (up to 90 min after sunrise). There is hence significant variation in emergence times in our population, without being linked to any of our fitness proxies. Variation could be maintained via balancing selection [50], of which frequency-dependent selection is an example. Here the fitness consequences of a certain trait depend on the frequency of that trait within the population [22]. In the case of chronotypes, the benefits of being early may only outweigh possible costs, if the rest of the population is relatively late. Then an individual could, for example, forage before the others. This could lead to selection for earlier chronotypes until this advantage disappears when too many other individuals are early too. Additionally, depending on the environment in which an individual lives, selection pressures on the timing of activities may differ, for example, due to condition dependence, resulting in spatially or temporarily fluctuating selection pressures [23]. Selection pressures might also have changed, e.g. in the context of anthropogenic change and urbanization, which may among others alter food availability. Thus, we might observe responses to current, possibly novel selection pressures, rather than an outcome of a selective process.

Unfortunately, we could not investigate the differences in the relationship between chronotype and reproductive success and survival between years, due to convergence problems with the models. This would have enabled us to explore this possibility by linking it to among-year variation in environmental variables. However, even within populations, individuals may occupy different ecological niches and trait optima may thus differ among individuals based on their individual niche specialization (matching habitat choice [51,52]). One may have to manipulate environmental factors at the nest level in order to fully understand the adaptive nature. This could be shown in one of our earlier studies [43], and such trait correlation may constrain its evolution. Similarly, selection could differ between the sexes, as earlier timing of male dawn song is related to more extra-pair copulations and hence potentially fitness, while we did not find directional selection pressures on the timing of activity onset in females. Such constraining processes could maintain trait variation, but could not be investigated within the framework of this study.

## 5. Conclusions

Here, we studied the relationships between the timing of activity (here, emergence time) in free-living animals, longevity and both annual as well as lifetime reproductive success. These estimates are closely linked to an individual’s fitness and may hence allow to prove the adaptive significance of among-individual variation in chronotypes. However, we did not find any signs of stabilizing, directional or disruptive selection, so currently the eco-evolutionary implications of individual variation in chronotypes remain elusive. Given that our findings align with findings in several other populations and species, the observed patterns are, however, possibly generalizable. Nevertheless, further studies will be necessary to understand the adaptive nature of chronotypes. These studies should integrate relevant parameters of the environment at the individual level, as identification of trait optima could become blurred by phenotype habitat matching. Future studies should also consider possible differences in selection pressures between the sexes.

## Data Availability

All data that support the findings of this study are provided as supplementary material [53].
